# General Properties of Colon Polyps in Central Anatolia

**DOI:** 10.5005/jp-journals-10018-1088

**Published:** 2014-01-22

**Authors:** Ayse Kefeli, Sebahat Basyigit, Abdullah Ozgur Yeniova, Yasar Nazligul, Metin Kucukazman, Bora Aktas

**Affiliations:** 1 Department of Gastroenterology, Kecib’ren Education and Research Hospital, Turkey; 2 Department of Gastroenterology, Kecib’ren Education and Research Hospital, Turkey; 3 Department of Gastroenterology, Kecib’ren Education and Research Hospital, Turkey; 4 Department of Gastroenterology, Kecib’ren Education and Research Hospital, Turkey; 5 Department of Gastroenterology, Kecib’ren Education and Research Hospital, Turkey; 6 Department of Gastroenterology, Kecib’ren Education and Research Hospital, Turkey

**Keywords:** Colorectal polyps, Colonoscopy, Gastroenterology.

## Abstract

**Aim:**

Polyp is called formations protruding into the lumen in the gastrointestinal tract. In this study, we aimed to determine frequency, anatomic distribution within the large bowel and pathologic types of colon polyps based on the colonoscopy results.

**Materials and methods:**

The colonoscopy reports between 2010 and 2011 were analyzed retrospectively. Complaint of the patients on admission, the presence of polyps, anatomic distribution of polyps, polyp type and polyp size were evaluated.

**Result:**

A total of 4000 colonoscopy reports were examined. The largest proportion of polyps occurred in the left colon (74.4%), followed by the ascending colon (11.5%). Eighty-five were adenomatous polyps, 71 were hyperplastic polyps, 12 were inflammatory polyps, 12 were serrated adenoma, 3 were adenocarcinoma, and 8 were *tubulovillous* adenomas.

**Discussion:**

In light of like this study, the development of screening and follow-up programs in Turkey will be useful to reduce the incidence and mortality of colorectal cancer.

**How to cite this article:** Kefeli A, Basyigit S, Yeniova AO, Nazligul Y, Kucukazman M, Aktas B. General Properties of Colon Polyps in Central Anatolia. Euroasian J Hepato-Gastroenterol 2014;4(1):7-10.

## INTRODUCTION

Polyp presents formations of protruding lesions into the lumen of the gastrointestinal tract. In other words, luminal protrusion or elevation of mucosal surfaces are called polyp.^1^ In the literature, the first cases of polyps have been reported about 300 years ago. Polyp can be asymptomatic; however, they also can cause abdominal pain, anemia, bleeding or obstruction. Polyps, especially to those who adenomatous types, may become malignant. For this reason, polyps detected during colonoscopy, regardless of size or type, are considered as the precursor lesion of colorectal cancer and their removal is recommended.

Polyp is thought to occur as a result of the failure or inability of proliferation, differentiation or apoptosis of normal cell in the colonic mucosa. Polyps are classified according to macroscopic appearance, size, number, anatomic distribution and histology. According to the histological characteristics of polyps, they are classified as nonneoplastic, neoplastic and submucosal lesions. The vast majority of polyps are smaller nonneoplastic polyps. Due to the high malignant potential of neoplastic polyps, they should be removed and underwent specific follow-up program.

As a result of advances in endoscopic procedures, endoscopic removable of polyps and definable of histologic types of polyps increased the interest in polyp. It has become possible to prevent the development of colon cancer and follow-up patients with this approach.

The frequency of polyps detected during endoscopy and at autopsy shows that regional and social differences may play a role. In this study, we aimed to determine frequency and anatomic distribution, histopathologic types of colon polyps based on the colonoscopy results.

## MATERIALS AND METHODS

The colonoscopy reports of patients who underwent colonoscopy for various reasons between the years of 2010 and 2011 in The Kecioren Training and Research Hospital, Ankara, Turkey were analyzed retrospectively. The age and sex distribution of the patients, complaint of the patients on admission, the presence of polyps, anatomic distribution of polyps, polyp type and polyp size were also evaluated. In a total of 4000 patients who had undergone colonoscopic examination, patients with colon polyps were analyzed. Patients were excluded who have diagnosis or a history of colorectal cancer or adenomatous polyps, significant obstructive lesions, family history of familial colorectal cancer syndrome and inflammatory bowel disease.

## STATISTICAL ANALYSIS

Data were analyzed by using a commercially available statistics software package (SPSS for Windows v. 15.0, Chicago, IL, USA). Categorical variables were expressed as the number (n) and percentage (%) and the chi-square test was used for the analysis. Results are presented as mean ± standard deviation and percentages.

## RESULT

Four thousand colonoscopy reports were examined and a total of 192 colon polyps were observed in 166 patients. Of these, 71 (42.8%) were male and 95 (57.2%) were female. The mean age of the patients was 60.14 ± 12.92 and the oldest patient was 81 years old, while the youngest patient was 21 years old.

**Graph 1:**
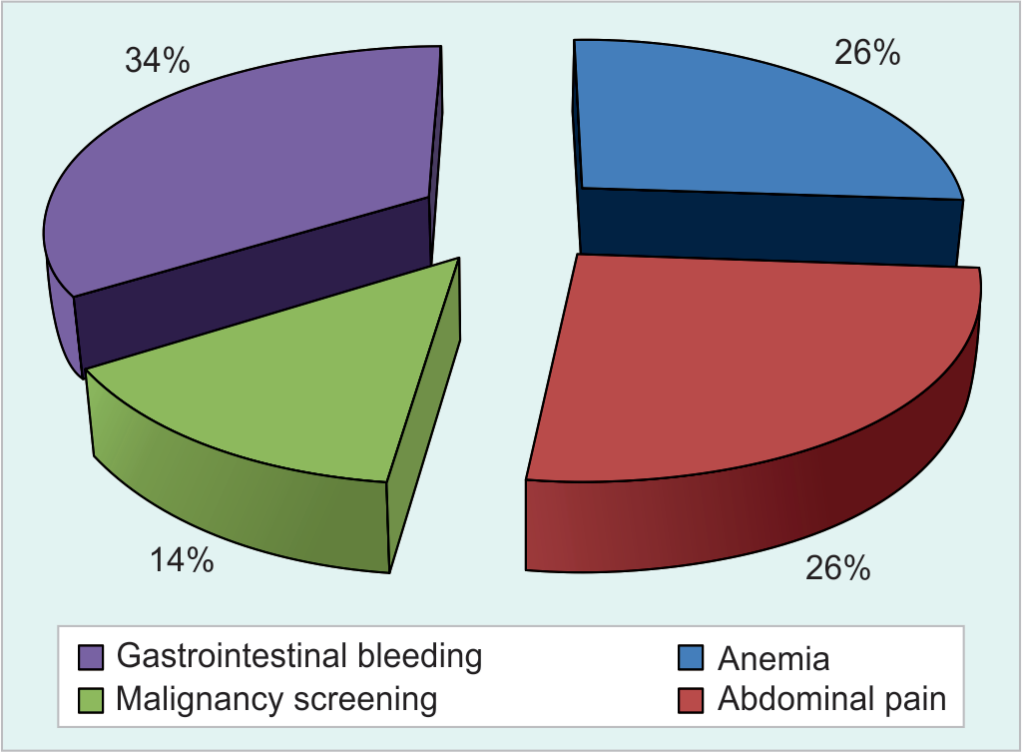
Causes of indications for colonoscopy

The most frequent cause of indications for colonoscopy is anemia (25.9%) and abdominal pain (25.9%), followed by the malignancy screening (13.9%), gastrointestinal bleeding (33.7%) is the most common cause if evaluating together anemia and hematochezia (Graph 1).

**Graph 2:**
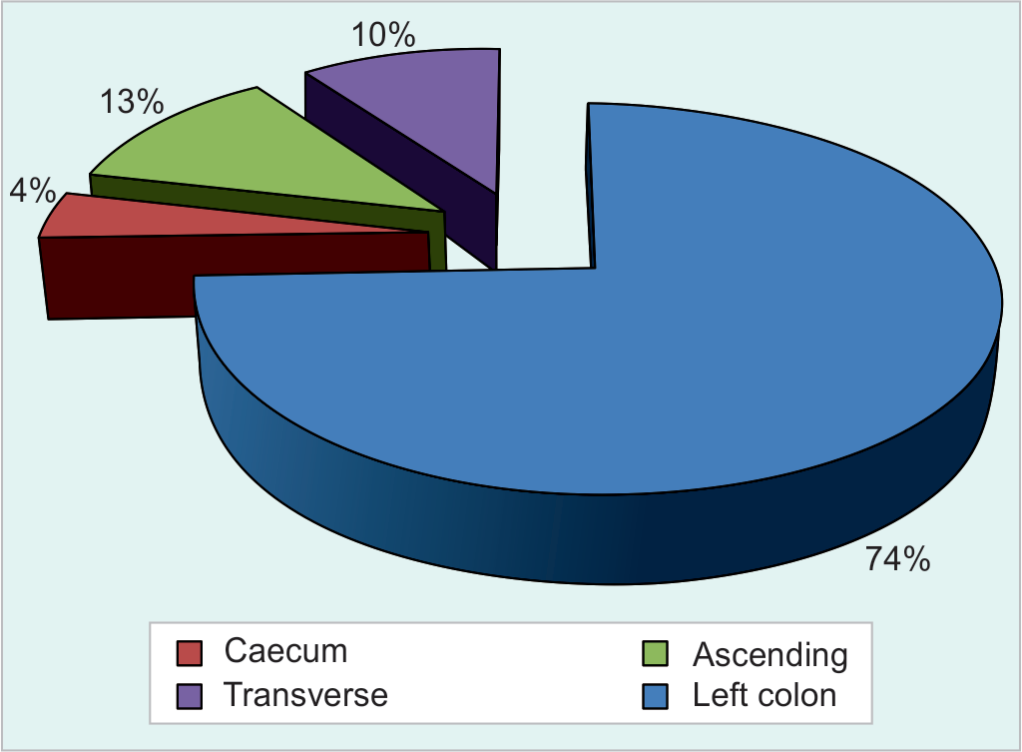
The topographical distribution of colon polyps

The largest proportion of polyps occurred in the left colon (74.4%), followed by the ascending colon (11.5%) and transverse colon (9.9%). The least number of polyps was seen in the cecum (4.2%) (Graph 2). Adenomatous polyps were present often in left colon (85.8), while 119 patients (71.7%) of the cases had a single polyp and 47 (28.3%) patients had polyps in multiple locations.

**Graph 3:**
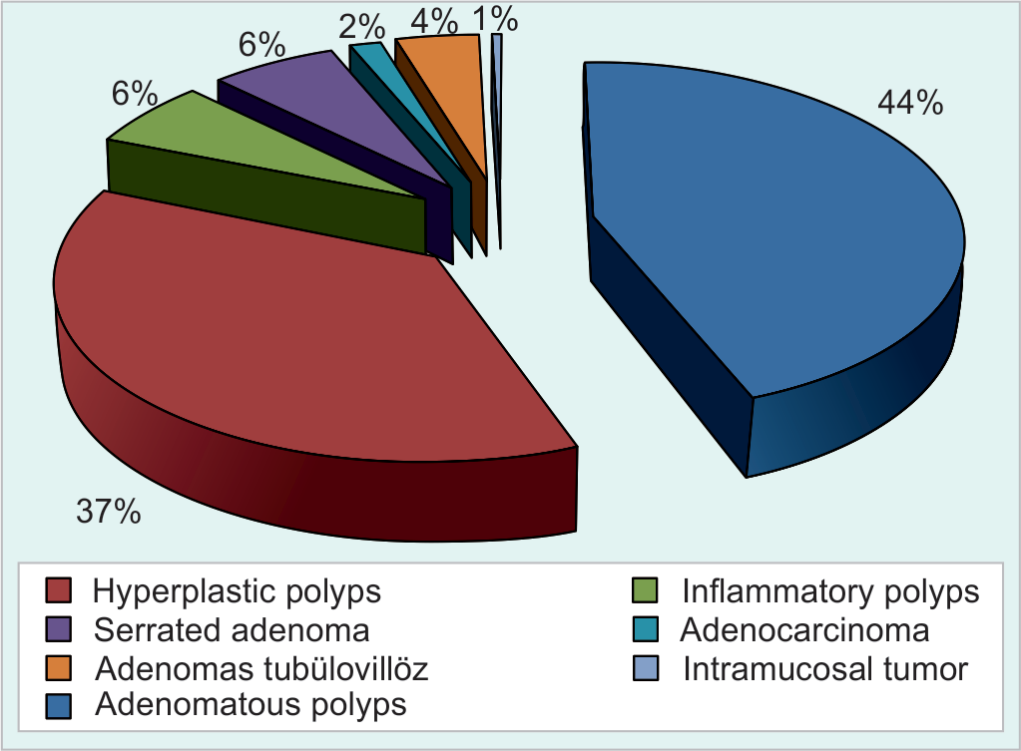
Types of polyps according to histopathology

The average polyp diameter was 8.92 mm (3-41 mm). A total of 151 (78.6%) polyps were within 10 mm in diameter, whereas 41(21.4%) of them were over 10 mm in diameter. Of these 68.2% were described as diminutive in size in endoscopic appearance. Eighty five (44.3%) polyps were adenomatous polyps, 71(37%) were hyperplastic polyps, 12 (6.3%) were inflammatory polyps, 12 (6.3%) were serrated adenoma, 3 (1.3%) were adenocarcinoma, 8 (4.2%) were adenomas tubulovillous, 1 (0.5%) was intramucosal tumor of all these polyps (Graph 3).

## DISCUSSION

Gastrointestinal polyps are seen more frequently in the colon. Most of them are multiple and often composed in the rectosigmoid region and frequency of the appearance is reduced toward the cecum. The largest proportion of polyps occurred in the left colon (74.4%), followed by the ascending colon (11.5%) and transverse colon (9.9%). The least number of polyps was seen in the cecum (4.2%) and that data are consistent with the literature.

Colorectal polyps may be symptomatic or asymptomatic. While they are symptomatic, they cause mostly rectal bleeding. They can cause abdominal pain, constipation or bowel obstruction. The most important clinical sign suggestive of polyp is rectal bleeding. Polyps are among the causes of rectal bleeding by 20%. Bleeding is seen more common in polyps larger than 1 cm. Fecal occult blood is positive with rate of 40 to 60%. Around 1 to 3% of asymptomatic people over the age of 40 are positive for fecal occult blood. The adenomas have been found in 30 to 35% of these and cancer has been found in 8 to 12% of these. In this study, approximately 10% of the cases presented with rectal bleeding, but some of the cases frequently ignores this complaint because of the social and psychological reasons was observed.

Colorectal polyps can be stalked or sessile. The sizes of polyps are variable. Colorectal polyps usually grow slowly. To reach 1 cm from 0.5 cm in diameter should be 2 to 3 years for a polyp and to turn into cancer should be 2 to 5 years for 1 cm polyp.^2-4^ Diminutive polyps are 5 mm or under in diameter and often seen in endoscopic procedure but their biological or clinical importance is less and they do not contain invasive carcinoma.^5,6^ In this study, it was detected that diminutive polyps were found to be the most common. When the distal polyps are larger than 5 mm, the possible presence of proximal adenomas increases.^7^

The classification of polyps is based on histological types. Histological structures of polyps determine the malignant potential.^8^ Therefore, polyp detected radiologically or endoscopically should be removed, regardless of the diameter and the histological type and clinical behavior of the polyp should be enlightened.

Colorectal polyps are divided into two main classes as neoplastic and nonneoplastic polyps. The most common type of nonneoplastic polyps are hyperplastic polyps. In this study, 37% of all colon polyps and 74% of nonneoplastic polyps were hyperplastic polyp. The size of hyperplastic polyps is mostly 5 mm in diameter or under. However, it is reported that in the presence of hyperplastic polyps in some circumstances indicate increased risk of cancer. Genetic studies heve presented that specific genetic changes could be seen (e.g. Microstellite instability) in hyperplastic polyps larger than 10 mm in diameter particularly in the right colon, and this kind of hyperplastic polyps had increased the risk of cancer development. Therefore, the approaches that ‘if there were hyperplastic polyps especially located in the left column it could be called normal colonoscopic assessment’ or ‘if there were hyperplastic polyps, it was not needed to follow-up’ are no longer valid.

Adenomatous transformation occurs in 13% of hyper-plastic polyps. It is called that mixed adenomatoushyper-plastic polyp. While they have structure of hyperplastic polyps in microscopic examination they show atypia at the cellular level. These are called serrated adenomas and they form a subset of hyperplastic polyp that not fully distinguished yet. These lesions may be converted to cancer. Thus, the view has appeared that hyperplastic lesions were not completely innocent.^9-11^ In this study, consistent with the literature, 12.9% of all hyperplastic polyps were defined as serrated adenoma. Adenomatous polyps of which neoplastic polyps create the most clinically important group of polyps detected by colonoscopy. They occupy also about 2/3 part of the all colon polyps. In this study, it is identified that the adenomatous polyps were 48.5% of the entire colon polyps. The majority of adenomas are tubular adenomas. Pure villous adenomas are very rare. According to the World Health Organization, adenomas are classified as tubular adenoma, if at least 80% of glands is branched or as villous adenomas, if at least 80% of glands is villiform.^12^ The most common form of adenomatous polyps is tubular adenomas (80%). Villous structure, large size, number of polyps and high-grade dysplasia are directly proportional to risk of malignancy.^13^ Age is a key factor in development and the frequency of adenomas. The incidence of polyps, multiple polyps probability and prevalence of dysplasia increase are correlated with age. ^2,14^

When the polyp is seen, it should be completely removed. In addition, a negative result in biopsy material obtained from polyp does not exclude cancer. Total excision of polyps is needed for exact histological diagnosis. An adenomatous polyp should be considered as a marker of colon cancer. Detection, removal and follow-up of polyps by colonoscopy are crucial to prevent the development of cancer. Because of routine removal of polyps found on sigmoidoscopy, the prevalence of cancer in this group of patients has decreased by 85%. There has not been a study on a large scale in Turkey to determine the prevalence of colorectal polyps.

In this study, the prevalence of various types of colon polyps revealed in our patients undergoing colonoscopy with the various indications. The data in the literature have shown that more frequent incidences of polyps occur in men in the population containing an average of 50 years age group; the left colon is the primarily placement of polyps; adenomas constitute histologically the largest percentage of polyps; the most common cause of admission is the rectal bleeding. In our study, data are consistent with the literature.

Large scale and prospective studies are needed to determine the prevalence of polyps with dysplasia. In light of these studies, the development of screening and follow-up programs in our country will be useful to reduce the incidence and mortality of colorectal cancer.
